# Preventive efficiency of neuromuscular training in reducing lower limb injuries among youth soccer athletes: a systematic review and meta-analysis of randomized controlled trials

**DOI:** 10.3389/fspor.2026.1853354

**Published:** 2026-07-08

**Authors:** Hussain Mohammed Alkishnawi, Hamza Mahmoud Alshater, Eyad A. Alakkas, Nawaf Dhafer Alshehri, Norah Adel Alsubaya, Naser B. A. J. Alenezi, Abdullah Eid Alatawi, Ahmad Basheer Basheer, Ibrahim Saleh Allehaimeed, Assad Mohammed Almaghrabi, Muhannad Algarni

**Affiliations:** 1 Physical Therapy Department, Royal Commission Medical Center, Yanbu, Saudi Arabia; 2 Department of General Surgery and Orthopedic Surgery, Faculty of Medicine, Ibn Sina National College, Jeddah, Saudi Arabia; 3 Department of Surgery/Orthopedic Section, King Faisal Specialist Hospital & Research Center, Jeddah, Saudi Arabia; 4 Faculty of Medicine, King Khalid University, Abha, Saudi Arabia; 5 Physical Therapy Department, Heraa General Hospital, Makkah, Saudi Arabia; 6 New Jahra Hospital, Ministry of Health, Kuwait City, Kuwait; 7 Faculty of Medicine, Tabuk University, Tabuk, Saudi Arabia; 8 Faculty of Medicine, Jordan University of Science & Technology, Irbid, Jordan; 9 General Practice, Qassim Health Cluster, Ministry of Health, Buraydah, Saudi Arabia; 10 Faculty of Medicine, King Abdulaziz University, Jeddah, Saudi Arabia

**Keywords:** ankle injury, injury prevention, meta-analysis, neuromuscular training, youth soccer

## Abstract

Lower-limb injuries are common in youth soccer. Neuromuscular training (NMT) warm-ups are used, but the preventive effects across injury types in young players are unclear. This review quantified NMT effectiveness for lower-limb injury prevention in 8–18-year-old soccer players. A PRISMA-guided systematic review and meta-analysis of randomized controlled trials was conducted. Four databases were searched up to October 2025 using keywords including soccer/football, neuromuscular training, balance, proprioception, plyometrics, injury prevention, ACL, ankle sprain, and hamstring strain. English-language RCTs (January 2000-present) in youth soccer comparing NMT with standard warm-up or no intervention were included; adult (>18 years), non-soccer, and non-RCT studies were excluded. The primary outcome was the incidence of all lower-limb injuries; secondary outcomes were knee, ankle, ligament, and muscle injuries. Incidence rate ratios (IRRs) were pooled using random-effects models with the Hartung–Knapp adjustment for outcomes in which at least two trials reported exposure time; the remaining outcomes were summarised using a structured narrative synthesis. Heterogeneity was assessed with I^2^. Five RCTs (2012–2025; *n* = 6,447) were included. Two outcomes had sufficient exposure data for meta-analysis: NMT was associated with non-significant reductions in all lower-limb injuries (IRR = 0.54, 95% CI 0.20–1.45; I^2^ = 83.7%) and knee injuries (IRR = 0.86, 95% CI 0.62–1.19; I^2^ = 0%). For ankle, ligament and muscle injuries only one trial reported exposure time, precluding pooling; in that trial NMT was associated with significantly lower ankle (IRR = 0.59, 95% CI 0.40–0.88), ligament (IRR = 0.62, 95% CI 0.43–0.90) and muscle (IRR = 0.66, 95% CI 0.47–0.93) injury rates, with the remaining trials reporting fewer injuries in the NMT group. NMT warm-ups may reduce ankle injuries in youth soccer, while evidence for overall and knee injury prevention is inconclusive and heterogeneous. Implementation with adherence support is clinically relevant. Future trials should standardize injury definitions, report exposure/compliance, and evaluate programme dose and components across age and sex groups.

**Systematic Review Registration:**

https://www.crd.york.ac.uk/PROSPERO/view/CRD420251207720, identifier CRD420251207720

## 1 Introduction

Organized soccer is a cornerstone of youth sport, but the speed of play, frequent cutting, and repeated jump landings expose young athletes to a high burden of lower-limb injury. Hamstring strains, ankle sprains, and anterior cruciate ligament (ACL) injuries are consistently reported as common and function-limiting, and injury severity and time-loss tend to increase with maturation (1, 2). Injuries during youth can have long-term consequences, including reduced future physical activity and broader health impacts, so prevention is a priority for coaches, clinicians, and sporting organizations (2). Neuromuscular training (NMT) warm-ups are widely promoted exercise-based strategies that combine balance, strength, plyometrics, proprioception, and agility to improve movement quality, postural stability, and neuromuscular control, thereby targeting modifiable risk factors for non-contact and overuse lower-limb injuries (1).

Evidence for NMT in soccer is drawn largely from cluster-randomized trials that integrate structured warm-ups into routine team sessions. In adolescent female players (13–17 years), FIFA 11+ (about 20 min per session, several times weekly) was associated with lower overall injury risk and clearer reductions in overuse and severe injuries (1). In Canadian players (13–18 years), adding neuromuscular exercises and home-based balance training to a standard warm-up reduced all-injury rates compared with usual practice (1). Programmes emphasizing knee and trunk control may be particularly relevant for ACL prevention. A large Swedish trial in female adolescents (12–17 years) tested a neuromuscular warm-up focused on knee control and core stability and reported a marked reduction in ACL injury rate (1). Trials also indicate that delivery matters. In one cluster trial, enhanced implementation support was associated with substantially lower injury incidence than controls, whereas a less intensive delivery strategy produced smaller effects (1). Evidence in male youth players suggests similar potential. In Nigerian youth soccer, FIFA 11+ was associated with reduced overall injury risk and a clearer reduction in lower-limb injuries (1). However, findings are not uniform. A German cluster trial comparing shorter and longer versions of a warm-up programme reported no meaningful reductions in overall injury incidence or key lower-limb subcategories, underscoring the possible role of programme dose, content, and setting (1).

The evidence base is thinner in children younger than 14 years despite the practical importance of this group in grassroots and academy soccer. The literature notes that few trials have evaluated soccer-specific NMT warm-ups in children, including FIFA 11+ Kids, and these studies reported large relative reductions in overall injury incidence compared with standard warm-ups (2). Hilska and colleagues addressed this gap in competitive U11–U14 players by replacing usual warm-up activities with an NMT warm-up performed 2–3 times per week over 20 weeks. The intervention did not significantly change the rate of all acute lower-limb injuries, but it reduced acute non-contact lower-limb injuries and also reduced ankle and joint or ligament injury subcategories (2). This distinction is clinically relevant because all-injury outcomes can dilute programme effects when interventions primarily target non-contact mechanisms (2). Adherence appears central to real-world impact. In the Finnish cohort, teams averaged about 1.7 sessions per week and adherence declined across the season; analyses restricted to high-adherence teams and an efficacy subset showed a stronger protective effect on non-contact lower-limb injuries (3). Similar seasonal deterioration has been reported in large adolescent NMT implementations, where both team-level completion and player-level compliance decreased as the season progressed (4). Coaches also identify practical barriers, including programme duration, limited space or time, and concerns about age-appropriateness, which can hinder sustained fidelity outside controlled research settings (3).

Systematic reviews and meta-analyses increasingly focus on ACL injury. A recent soccer-specific meta-analysis reported that injury-risk-reduction programmes including balance training reduce ACL injury rates, with benefits in males and females (5). Earlier meta-analytic work in female athletes also supported neuromuscular preventive programmes and suggested stronger effects in athletes younger than 18 years and in soccer (6). Uncertainties remain regarding which components drive protection (for example, balance-dominant vs. plyometric and strength-dominant approaches), the minimum effective dose, and whether benefits extend consistently beyond ACL to other common lower-limb injuries such as ankle sprains and muscle strains (5, 6). Many syntheses prioritize ACL outcomes, mix adolescents with adults, or include non-randomized designs, so soccer-specific estimates for players aged approximately 8–18 years remain needed. The limited paediatric trials further suggest that benefits may be concentrated in non-contact injuries and may depend heavily on adherence, which commonly declines during a season (2–4). Therefore, this systematic review and meta-analysis evaluated the preventive effectiveness of NMT programs for reducing lower-limb injuries in youth and adolescent soccer players. We aimed to identify randomized controlled trials comparing NMT-based warm-ups or training programs with standard warm-ups, traditional training, or no intervention; pool exposure-adjusted effects for outcomes with sufficient data; summarize injury-specific outcomes narratively where pooling was not possible; and assess study-level risk of bias to inform interpretation of the evidence. Accordingly, we hypothesized that NMT programs would reduce the incidence of lower-limb injuries among youth soccer players.

## 2 Methods

### 2.1 Review of the literature

**Figure 1:**
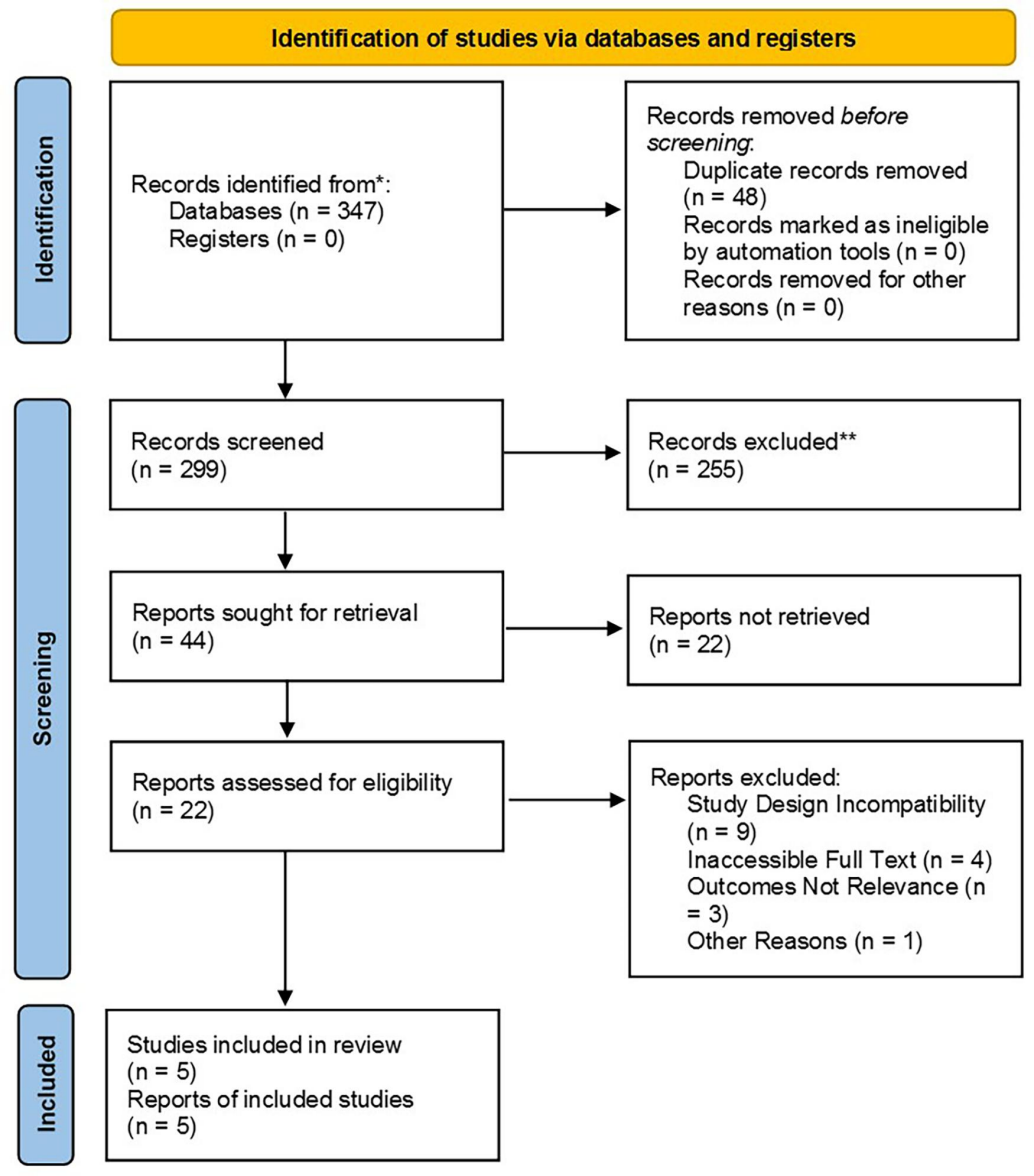
PRISMA 2020 flow diagram of study selection.

For this systematic review, we followed the PRISMA (Preferred Reporting Items of Systematic Reviews and Meta-Analysis) model to ensure that studies were selected with the least amount of bias (7). The flow diagram summarizing identification, screening, eligibility, and inclusion of studies is presented in Figure 1. This study protocol was registered with PROSPERO *a priori* with the following ID: CRD420251207720 (8). A systematic search of PubMed, Google Scholar, Cochrane, and Embase was conducted in October 2025. A search was conducted using the following keywords: (Soccer OR football OR soccer players) AND (neuromuscular training OR NMT OR balance training OR proprioceptive training OR stability exercises OR plyometric training) AND (injury prevention OR lower limb injury OR ACL injury OR ankle sprain OR hamstring strain). Database-specific search strings, filters, and date limits are provided in Supplementary Table 1. Based on PICOS (population, intervention, comparison, outcome, study design) criteria, studies were considered for the review (9).

### 2.2 Methodology for selecting studies

For inclusion in our systematic review, the studies had to meet the following criteria: (1) they had to be in the English language, (2) they had to be published between January 2000 and the present, (3) the participants had to be youth soccer players aged 8–18 years, (4) they had to be undergoing Neuromuscular Training (NMT) programs (including components such as balance, agility, strength, neuromuscular control, proprioception, (5) They had to be athletes with reported lower limb injuries, such as ACL injuries, ankle sprains, and hamstring strains, and (6) the studies had to be randomized controlled trials (RCTs) to be included in the review.

Studies were excluded from our systematic review based on the following criteria: (1) studies published before January 2000, (2) English was not the language of study, (3) adults soccer players, older than 18 years, (4) Individuals undergoing non neuromuscular intervention, and (5) the following study types: prospective cohort, retrospective cohort, cross-sectional, case reports, or any other type except RCTs.

### 2.3 Process of screening and data extraction

Two independent reviewers (HK and HS) screened papers simultaneously and independently reviewed them by title and abstract using the Rayyan search web for systematic reviews (10). Then, the full text of the articles were reviewed by two independent reviewers (AB and AA) simultaneously, with any differences being resolved by a third reviewer (NS). After which, data extraction was performed by a reviewer (NS) for the following variables: (1) Total number of patients (2) Number of patients in Group 1 (NMT) (3) Number of patients in Group 2 (NON-NMT) (4) Outcomes being measured (5) Age range of patient (6) Mean Age in years, SD (7) Number of patients with male sex (8) Number of patients with female sex (9) Type of sport (10) Follow up duration (11) Program timing (12) Type of intervention (13) Type in comparison group (14) Frequency (15) Duration (16) All lower limb injuries (17) Knee injuries (18) Ankle injuries (19) Ligament injury (20) Muscle injuries. To avoid duplication, the retrieved data were double-checked.

Exposure data were extracted as athlete-exposure hours when reported in the published articles. When outcome-specific exposure time was not reported, the available total athlete-exposure time for the corresponding trial arm was extracted if applicable. Missing or unreported values were coded as “NR” (not reported). Study authors were not formally contacted for additional unpublished data or clarification of missing exposure hours. Therefore, outcomes for which fewer than two trials reported both injury events and exposure time were not pooled quantitatively and were instead summarized narratively.

### 2.4 Assessment of quality and bias risk

**Figure 2:**
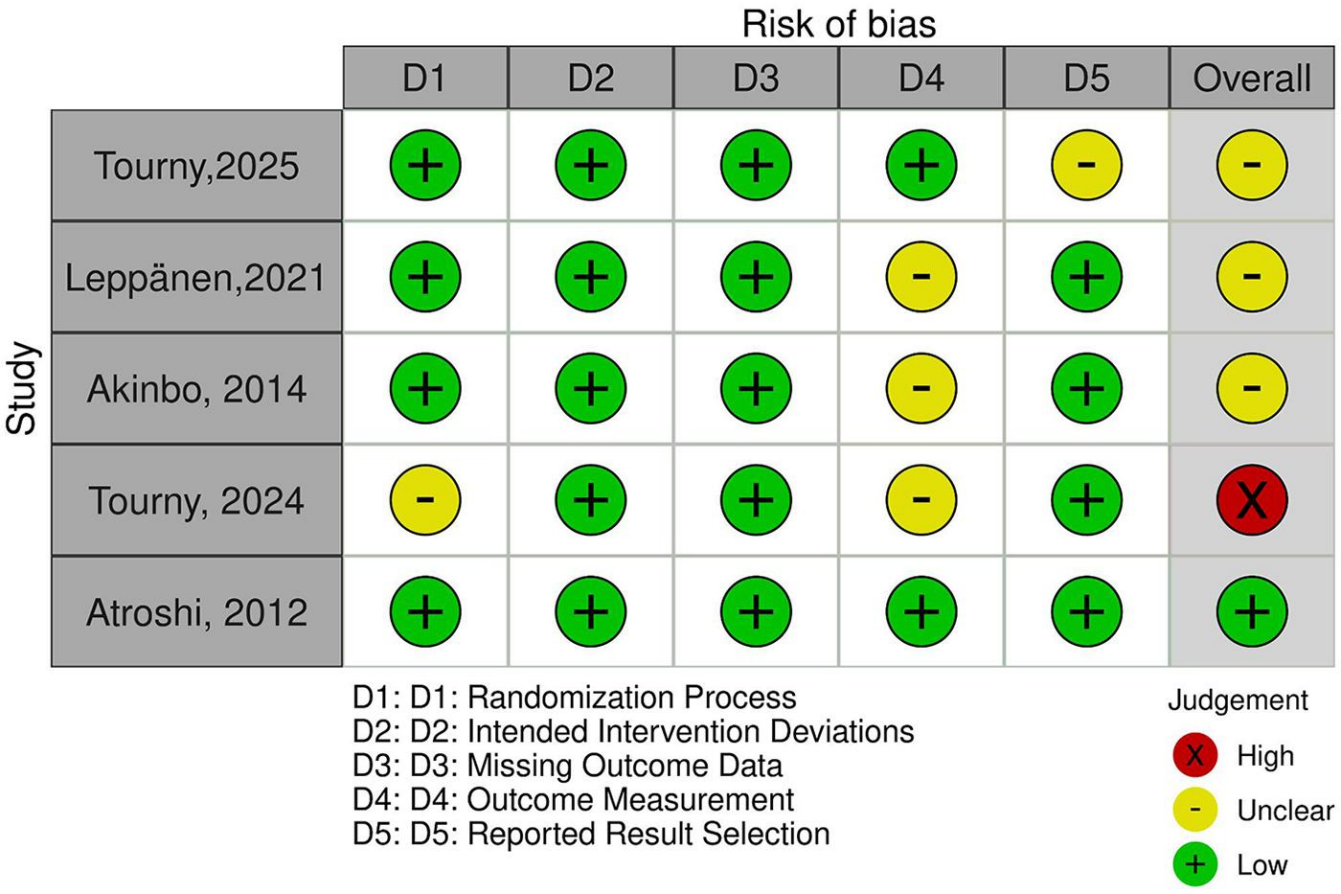
Risk-of-bias assessment of the included randomized controlled trials.

The risk of bias for each included study was assessed using the appropriate tool based on its design. We used the Cochrane Collaboration's Risk of Bias (RoB) 2 tool (11) for Randomized Controlled Trials (RCTs) to evaluate five domains: bias arising from the randomization process, bias due to deviations in intended interventions, bias due to missing outcome data, bias in measurement of the outcome, and bias in selection of the reported result. Two independent reviewers (NA and MG) assessed each study's risk of bias, resolving disagreements through discussion or by consulting a third reviewer (HK). The overall risk of bias for each study was classified as low, high, or unclear based on the assessment of these domains (Table 1, Figure 2). No formal outcome-level certainty-of-evidence assessment, such as GRADE, was performed; therefore, the assessment was limited to study-level risk of bias using the RoB 2 tool.

**Table 1:** Bias assessment for included studies.

Study ID	Authors/year	Study type	D1: randomization process	D2: intended intervention deviations	D3: missing outcome data	D4: outcome measurement	D5: reported result selection	Overall risk
S001	Tourny, 2025	RCT	Low	Low	Low	Low	Moderate	Moderate
S002	Leppänen, 2021	RCT	Low	Low	Low	Moderate	Low	Moderate
S003	Akinbo, 2014	RCT	Low	Low	Low	Moderate	Low	Moderate
S004	Tourny, 2024	RCT	Moderate	Low	Low	Moderate	Low	High
S005	Waldén, 2012	RCT	Low	Low	Low	Low	Low	Low

### 2.5 Meta-analysis of the included data

All analyses were performed in RStudio using the meta and metafor packages. Because the included trials counted recurrent injury events together with athlete-exposure time (player-hours), and because the number of events exceeded the number of participants in some trials, incidence rate ratios (IRRs) rather than risk ratios were used as the effect measure for the quantitative synthesis. For each outcome, the natural-logarithm IRR and its sampling variance were derived from the event counts in the two arms, and IRRs were pooled using a random-effects model fitted by restricted maximum-likelihood (REML), with confidence intervals and significance tests obtained using the Hartung–Knapp adjustment to account for the small number of trials; where only two trials contributed, and between-study heterogeneity was negligible, a common-effect estimate is also reported. Statistical heterogeneity was quantified using the I^2^ statistic and Cochran's *Q* test. A quantitative meta-analysis was undertaken only for outcomes in which at least two trials reported both injury events and exposure time, namely all lower-limb injuries and knee injuries. For ankle, ligament, and muscle injuries, exposure time was available from a single trial only; these outcomes were therefore summarised using a structured narrative synthesis, reporting incidence rates where exposure was available and event counts with the direction of effect otherwise. Because fewer than ten trials contributed to any outcome, funnel-plot–based assessment of publication bias was considered uninformative and was not performed.

## 3 Results

Our review included five RCTs, which were conducted between 2012 and 2025 across diverse geographic regions such as Europe, Africa, and North Africa. The sample sizes of included studies ranged from small experimental cohorts of 24 participants (Tourny, 2024) to large population-based trials that involved 4,564 youth soccer players (Waldén, 2012). Notably, more than 6,400 participants were included across all studies, and there is almost balanced allocation between NMT and control groups in most trials. All the included studies employed neuromuscular training-based interventions and compared them with the standard warm-up routines or traditional soccer training. The primary outcomes focused on injury prevention, with specific emphasis on the overall lower-limb injuries (Leppänen 2021, Akinbo 2014) and injuries of anterior cruciate ligament (Waldén, 2012). Some smaller trials also explored combined outcomes that are related to physical fitness and injury reduction (Tourny 2024, Tourny 2025) (Table 2).

**Table 2:** Characteristics of included randomized controlled trials.

Author	Journal	Country	Study design	Total sample (*N*)	NMT group (*n*)	Control/non-NMT (*n*)	Primary outcome
Tourny (2025)	Sports Medicine—Open	Morocco	RCT	40	20	20	Injury Prevention
Leppänen (2021)	Orthopaedic Journal of Sports Medicine	Finland	RCT	1,403	673	730	Soccer-related Acute Lower-limb Injury
Tourny (2024)	Sports Medicine—Open	Morocco	RCT	24	12	12	Physical Fitness and Injury Prevention
Akinbo (2014)	Journal of Sports Science and Medicine	Nigeria	RCT	416	212	204	Acute Lower-limb Injuries
Waldén (2012)	British Medical Journal	Sweden	RCT	4,564	2,479	2,085	Anterior Cruciate Ligament Injury

Table 3 shows the demographic characteristics and features of the neuromuscular training (NMT) program of the included randomized controlled trials. The majority of studies primarily involved youth and adolescent soccer players, with age ranges spanning from 9 to 18 years. Notably, the mean ages of participants were comparable between NMT and non-NMT groups within individual studies. Most of the studies were gender-specific as two trials included exclusively male participants (Tourny 2025, Akinbo 2014), while two focused solely on female athletes (Tourny 2024, Waldén 2012), and there is one large mixed-gender cohort (Leppänen, 2021). The duration of follow-up varied from 6 weeks to 7 months, with most of the studies implemented NMT programs across the full competitive season (Leppänen 2021, Waldén 2012). The shorter interventions were delivered during the preseason or in-season periods (Tourny 2024, Tourny 2025).

**Table 3:** Demographic and program characteristics reported in included studies.

Author	Age range	Mean age (Sd)	Male (*N*)				Female (*N*)			Sport	Follow-up duration	Program timing
		NMT	Non-NMT	NMT	Non-NMT	Total	NMT	Non-NMT	Total			
Tourny (2025)	NR	16.0 ± 0.62	17.5 ± 0.95	20	20	40	NR	NR	NR	Soccer	8 weeks	In-season
Leppänen (2021)	9−14 yrs	12.2 ± 1.2	12.3 ± 1.1	556	567	1,123	117	163	280	Soccer	20 weeks	Full season
Tourny (2024)	16−18 yrs	17.0 ± 1.3		NR	NR	NR	12	12	24	Soccer	6 weeks	Preseason
Akinbo (2014)	NR	17.80 ± 0.94	17.49 ± 1.10	212	204	416	NR	NR	NR	Soccer	6 months	Full season
Waldén (2012)	12−17 yrs	14.1 ± 1.2	14.0 ± 1.2	NR	NR	NR	2,479	2,085	4,564	Soccer	7 months	Full season

NR, Not reported; *N*, Number; SD, Standard deviation; NMT, Neuromuscular training.

Table 4 shows the structure and content of neuromuscular training (NMT) interventions implemented across the included randomized controlled trials. Although all studies evaluated NMT-based approaches, there was notable variability in the design of program, frequency, and duration. There are two studies which delivered NMT as a structured warm-up program, replacing or augmenting standard warm-up routines (Leppänen 2021, Waldén 2012). These interventions were relatively brief, which lasted 15–20 min per session, and were performed two to three times per week.

**Table 4:** Details of neuromuscular training interventions.

Author	Type of NMT intervention	Comparison group	Frequency		Session duration	
			NMT	Non-NMT	NMT	Non-NMT
Tourny (2025)	Neuromuscular training	Traditional soccer-specific training	2/week	2/week	60 min	60 min
Leppänen (2021)	NMT-based warm-up	Standard warm-up	2–3/week	NR	20 min	NR
Tourny (2024)	Neuromuscular training (general strengthening, plyometrics, agility, speed, core stability, balance, dynamic lumbo-pelvic control)	Endurance-dominated training	3/week	3/week	45–60 min	NR
Akinbo (2014)	FIFA 11+ warm-up program	Non-structured warm-up	2/week	NR	NR	NR
Waldén (2012)	NMT-based warm-up	Standard warm-up	2/week	NR	15 min	NR

NR, Not reported; *N*, Number; SD, Standard deviation; NMT, Neuromuscular training.

In contrast, there are some other trials which applied full-session NMT programs which incorporated into multiple components such as strength training, plyometrics, agility, balance, and core stability exercises (Tourny 2024, Tourny 2025). These programs were conducted two to three times weekly with the longer session durations of 45–60 min, which often matched in frequency to the comparison training to control for exposure. The FIFA 11+ warm-up program was a widely recognized injury-prevention protocol, was evaluated against a non-structured warm-up in one study (Akinbo 2014).

Table 5 shows the distribution of lower limb injury outcomes and corresponding exposure hours across the included randomized controlled trials. Most of the studies consistently reported fewer injuries in the NMT groups as compared to the non-NMT groups. For all lower limb injuries, a notable reduction was observed in trials with the larger samples and exposure-adjusted data, particularly in the study by Leppänen (2021), which reported 310 injuries in the NMT group as compared to 346 in the control group despite comparable exposure hours. Similar trends were evident in smaller trials, where NMT groups experienced substantially fewer total injuries than controls (Tourny 2024, Akinbo 2014).

**Table 5:** Lower limb injury outcomes and exposure across included studies.

Author	All LL injuries				
	NMT (*N*)	Exposure (h)	Non-NMT (*N*)	Exposure (h)	Total
Tourny (2025)	14	NR	28	NR	42
Leppänen (2021)	310	70,455	346	62,909	656
Tourny (2024)	21	4,485	50	4,485	71
Akinbo (2014)	26	51,017	76	61,045	102
Waldén (2012)	NR	NR	NR	NR	NR
Knee injuries					
Tourny (2025)	NR	NR	NR	NR	NR
Leppänen (2021)	21	70,000	25	64,103	46
Tourny (2024)	NR	NR	NR	NR	NR
Akinbo (2014)	12	NR	21	NR	33
Waldén (2012)	49	149,214	47	129,084	96
Ankle injuries					
Tourny (2025)		NR		NR	
Leppänen (2021)	40	71,429	60	63,158	100
Tourny (2024)		NR		NR	
Akinbo (2014)	10	NR	30	NR	40
Waldén (2012)	NR	NR	NR	NR	NR
Ligament injuries					
Tourny (2025)	2	NR	4	NR	6
Leppänen (2021)	48	70,588	69	63,303	117
Tourny (2024)	3	NR	14	NR	17
Akinbo (2014)	NR	NR	NR	NR	NR
Waldén (2012)	NR	NR	NR	NR	NR
Muscle injuries					
Tourny (2025)	1	NR	12	NR	13
Leppänen (2021)	57	71,250	77	63,636	134
Tourny (2024)	3	NR	12	NR	15
Akinbo (2014)	NR	NR	NR	NR	NR
Waldén (2012)	NR	NR	NR	NR	NR

NR, Not reported; *N*, Number; SD, Standard deviation; NMT, Neuromuscular training.

The knee injury related outcome included anterior cruciate ligament-related events, were reported in three studies and they showed comparable or reduced injury counts in NMT groups when adjusted for exposure (Leppänen 2021, Waldén 2012). Ankle, ligament, and muscle injuries were less frequently reported but they generally followed the same pattern, with lower injury counts among NMT participants (Leppänen 2021, Tourny 2024).

### 3.2 Meta-analysis and narrative synthesis of injury outcomes

**Figure 3:**
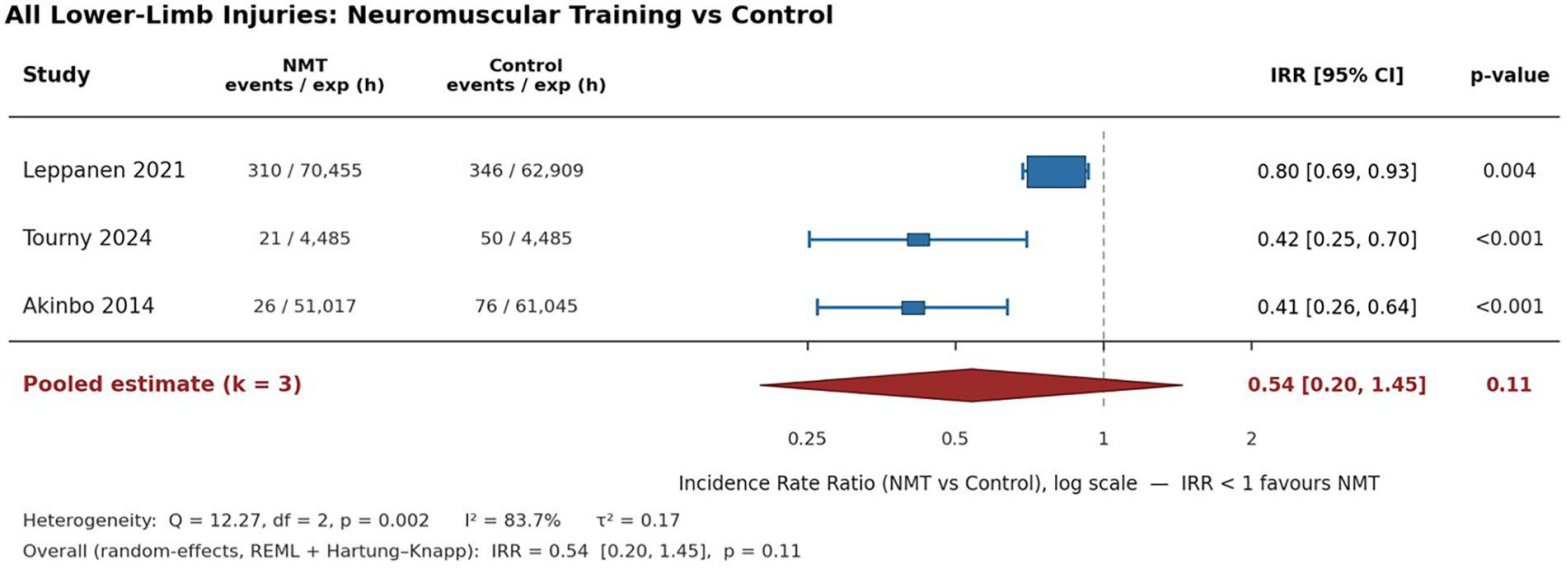
Forest plot of all lower-limb injuries comparing neuromuscular training with control.

Quantitative pooling was performed for the two outcomes with adequate exposure data, while the remaining outcomes were summarised narratively. For all lower-limb injuries, three trials (Leppänen 2021, Tourny 2024, and Akinbo 2014) reported both injury events and exposure time and were pooled. The random-effects model showed a non-significant reduction in the rate of all lower-limb injuries in the NMT group (IRR = 0.54, 95% CI 0.20–1.45, *p* = 0.11), with substantial heterogeneity (I^2^ = 83.7%; Q = 12.27, *p* = 0.002) (Figure 3). Although the pooled point estimate favoured NMT, the wide confidence interval, which crossed unity, reflected both the limited number of trials and the divergence between the largest trial (Leppänen 2021: IRR = 0.80, 95% CI 0.69–0.93) and the two smaller trials, which reported larger protective effects (Tourny 2024: IRR = 0.42, 95% CI 0.25–0.70; Akinbo 2014: IRR = 0.41, 95% CI 0.26–0.64).

**Figure 4:**
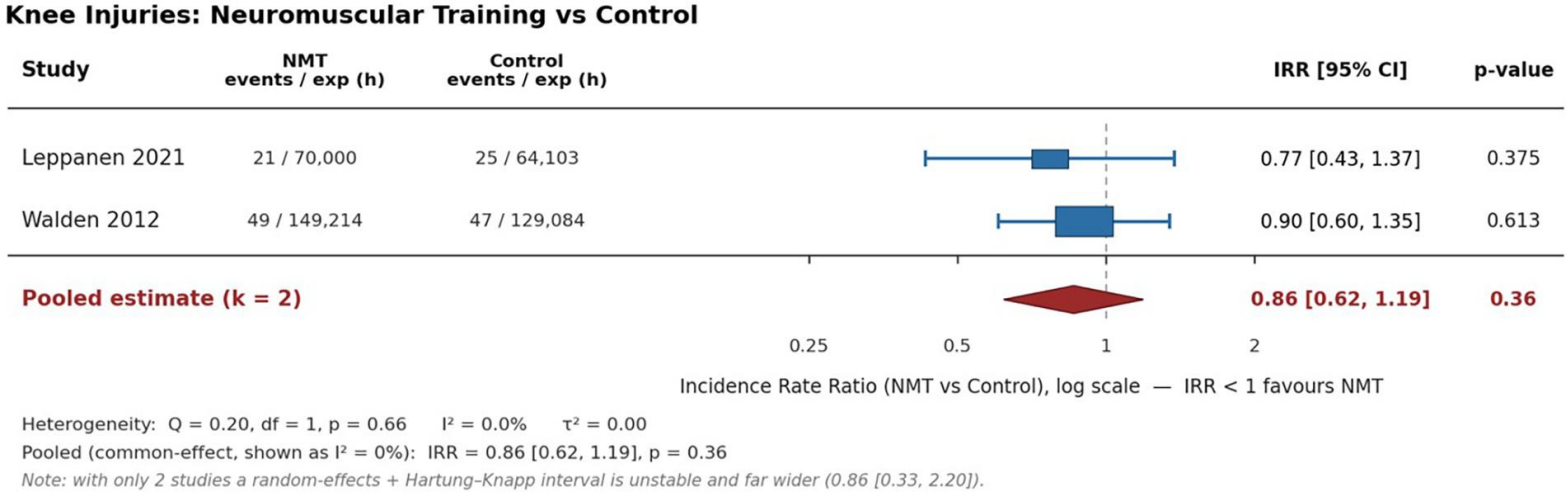
Forest plot of knee injuries comparing neuromuscular training with control.

For knee injuries, two trials (Leppänen 2021 and Waldén 2012) provided both events and exposure time. The pooled estimate showed no significant difference between groups (IRR = 0.86, 95% CI 0.62–1.19, *p* = 0.36) with no detectable heterogeneity (I^2^ = 0%; Q = 0.20, *p* = 0.66) (Figure 4). The two trials yielded consistent, near-null estimates (Leppänen 2021: IRR = 0.77, 95% CI 0.43–1.37; Waldén 2012: IRR = 0.90, 95% CI 0.60–1.35); because only two trials contributed, the common-effect estimate is reported, whereas the corresponding random-effects Hartung–Knapp interval was considerably wider and statistically unstable.

For ankle injuries, exposure time was available from a single trial. Leppänen (2021) reported a significantly lower ankle-injury rate in the NMT group (IRR = 0.59, 95% CI 0.40–0.88; 40 vs. 60 events), and Akinbo (2014) likewise reported fewer ankle injuries in the NMT group (10 vs. 30 events) but did not provide outcome-specific exposure data, precluding pooling. The available evidence was therefore consistent in direction, favouring NMT, but could not be quantitatively combined.

For ligament injuries, three trials reported injury counts, but only one provided exposure time. Leppänen (2021) reported a significantly lower rate in the NMT group (IRR = 0.62, 95% CI 0.43–0.90; 48 vs. 69 events), while the remaining trials reported fewer ligament injuries in the NMT group without exposure data (Tourny 2025: 2 vs. 4; Tourny 2024: 3 vs. 14). All available data favoured NMT, but the absence of exposure data in two of the three trials precluded pooling.

For muscle injuries, exposure time was again available only from Leppänen (2021), which reported a significantly lower rate in the NMT group (IRR = 0.66, 95% CI 0.47–0.93; 57 vs. 77 events). The other trials reported fewer muscle injuries in the NMT group without exposure data (Tourny 2025: 1 vs. 12; Tourny 2024: 3 vs. 12). The direction of effect consistently favoured NMT, although the data could not be pooled.

Because fewer than ten trials contributed to any outcome, formal assessment of publication bias using funnel-plot analysis was considered unreliable and was not performed.

## 4 Discussion

This systematic review and meta-analysis evaluated the potential preventive effect of neuromuscular training (NMT) programs on lower-limb injuries in youth soccer players. Across five randomized controlled trials including more than 6,400 participants, NMT was generally associated with injury estimates favoring the intervention; however, the strength and certainty of these findings varied by injury type, and most outcomes should be interpreted cautiously because of the small number of trials, incomplete exposure reporting, and non-significant pooled estimates.

### 4.1 Interpretation of main findings

Across outcomes, the most consistent protective signal was for ankle injuries. In the only trial that reported exposure time for this outcome, NMT was associated with a significantly lower ankle-injury rate (IRR = 0.59, 95% CI 0.40–0.88), corresponding to approximately a 41% lower incidence, and the second trial reporting ankle injuries also recorded substantially fewer events in the NMT group (10 vs. 30). Because only one trial provided exposure data, these results could not be pooled, and the finding should therefore be regarded as promising but preliminary. It nonetheless remains clinically relevant given the high frequency and recurrence of ankle sprains in youth soccer, and it is consistent with the proprioceptive and balance components that are central to most NMT programmes.

For the two outcomes that could be pooled, the effects were protective in direction but did not reach statistical significance. For all lower-limb injuries, the random-effects incidence rate ratio was 0.54 (95% CI 0.20–1.45), indicating a lower injury rate with NMT but with a confidence interval that crossed unity and substantial heterogeneity (I^2^ = 83.7%). This heterogeneity largely reflected the contrast between the largest trial, which showed a more modest effect, and the two smaller trials, which reported larger reductions, so the pooled estimate should be interpreted cautiously. For knee injuries, the pooled estimate lay close to the null (IRR = 0.86, 95% CI 0.62–1.19) with no detectable heterogeneity (I^2^ = 0%), and the two contributing trials were highly consistent. Together, these findings imply that, while NMT may plausibly reduce knee and overall lower-limb injuries, the current evidence does not demonstrate a statistically significant benefit, including for anterior cruciate ligament injuries.

Ligament and muscle injuries could not be pooled because only a single trial reported exposure time for each. In that trial, NMT was associated with significantly lower ligament (IRR = 0.62, 95% CI 0.43–0.90) and muscle (IRR = 0.66, 95% CI 0.47–0.93) injury rates, and the remaining trials consistently reported fewer such injuries in the NMT group. Although the direction of effect was uniformly protective, reliance on single-study estimates and on unadjusted event counts means these findings require confirmation in adequately powered trials that report standardized exposure data.

The substantial heterogeneity observed for all lower-limb injuries likely reflects important clinical and methodological differences across the contributing trials rather than random variation alone. First, the pooled estimate combined one large trial showing a more modest protective effect with two smaller trials reporting larger reductions, suggesting that small-study effects and differences in implementation context may have influenced the overall estimate. Second, participant age varied across trials, ranging from younger players aged approximately 9–14 years to older adolescent players around 17–18 years. These age differences may modify the effect of NMT because maturation status, coordination, neuromuscular control, training history, and injury mechanisms differ between pre-adolescent children and late adolescents. Third, the content and dose of NMT varied substantially. Some interventions were delivered as brief warm-up programs emphasizing balance, proprioception, and movement control, whereas others used longer multi-component programs incorporating plyometrics, strengthening, agility, core stability, and sport-specific conditioning. Such differences may affect injury subtypes differently, with balance-dominant programs plausibly having greater effects on ankle-related mechanisms, while more intensive strength- and plyometric-focused programs may be required to influence knee, ligament, or muscle injury risk. Therefore, the pooled all lower-limb estimate should be interpreted as an average across heterogeneous interventions and populations rather than as a single uniform treatment effect.

The risk-of-bias assessment also affects the interpretation of the pooled findings. Tourny 2024 was judged to have a high overall risk of bias, mainly due to concerns in the selection of the reported result, and it was a small trial reporting a large protective effect for all lower-limb injuries. Its inclusion may therefore have contributed to the substantial heterogeneity and may have inflated the apparent magnitude of the pooled effect. Accordingly, all lower-limb estimates should be interpreted cautiously.

### 4.2 Reference to relevant literature

The current systematic review of five RCTs partially aligns with previous literature. While a seven-study review reported reductions in lower limb, knee, and ankle injuries with multi-component NMT, our analysis showed significant decreases only for ankle injuries, with non-significant effects for other outcomes. Differences may reflect intervention type, program complexity, and study heterogeneity. Overall, multi-component NMT appears more effective than isolated or shorter protocols (12).

NMT, especially the FIFA 11+ program, effectively reduces soccer-related injuries, particularly in female players and settings with high adherence and coach education. Although narrative evidence suggests reductions in overall and knee injuries, meta-analytic findings, including ours, show non-significant effects for most lower limb injuries due to heterogeneity and limited statistical power. Consistent ankle injury reductions highlight NMT's preventive role for distal lower limb injuries (1).

### 4.3 Potential mechanisms underlying observed effects

Variations in program design and the particular training components employed may be the cause of differences in NMT's efficacy among injury types. The majority of studies focused on proprioceptive activities, dynamic movement control, and balance training as therapies to improve neuromuscular coordination at the ankle joint. By improving body awareness and postural control, these training components help athletes better manage the demands placed on the ankle during rapid changes of direction, jumping, and landing movements that frequently occur in soccer and are commonly associated with ankle sprain injuries.

On the other hand, more rigorous, extended, and biomechanically focused training may be necessary to prevent knee, ligament, and muscle problems, especially ACL injuries. The capacity of NMT therapies to reliably affect these damage processes may have been hampered by variations in program length, session length, exercise progression, and adherence between studies. Furthermore, NMT was administered in some trials as quick warm-up exercises, which would be effective to prevent ankle injuries but insufficient to cause the neuromuscular changes required to considerably lessen more complicated injury patterns.

### 4.4 Clinical and practical implications

The findings of this synthesis have practical applications for youth soccer. Without the need for extra equipment or longer training sessions, NMT was associated with lower ankle-injury rates when incorporated into routine training, especially during warm-up periods, although this signal derived from a single exposure-reporting trial and should be confirmed. On balance, NMT can reasonably be regarded as a low-cost and potentially useful injury-prevention strategy for coaches, medical professionals, and sport regulatory authorities, pending stronger pooled evidence.

Nevertheless, the lack of statistically significant improvements for other injury outcomes suggests that a standard NMT approach might not be adequate. NMT therapies may need to modify training intensity, activity choice, and program duration to maximize preventive effects, especially when addressing knee or muscular injuries. Achieving significant injury reductions may also need regular delivery and ongoing attention throughout the competitive season.

### 4.5 Strengths and limitations in context

A number of acknowledged limitations should be taken into consideration when interpreting the review's conclusions. Variability in study populations, intervention strategies, follow-up times, and injury classification is highlighted by significant heterogeneity across pooled analyses. Lastly, it was difficult to adequately evaluate causes of heterogeneity or do subgroup analysis due to the small number of studies included. Another limitation was the incomplete reporting of exposure data across injury subcategories. Although exposure-adjusted IRRs were used whenever both injury events and athlete-exposure hours were available, several trials did not report outcome-specific exposure time for ankle, ligament, or muscle injuries. Because study authors were not formally contacted for additional unpublished exposure data, these outcomes could not be pooled and were summarized narratively. This limits the precision of injury-specific conclusions and highlights the need for future trials to report injury events alongside standardized athlete-exposure hours for each injury category. Finally, the present review did not include a formal outcome-level certainty-of-evidence assessment, such as GRADE. As a result, confidence in the evidence was not rated separately for each outcome. Interpretation, therefore, relied on study-level risk of bias, statistical heterogeneity, precision of the pooled estimates, and completeness of exposure reporting. Future reviews with a larger evidence base should incorporate a formal GRADE assessment to provide outcome-specific certainty ratings. Publication bias could not be reliably assessed because fewer than ten trials were available for any outcome. Funnel-plot analysis was therefore not performed, as visual asymmetry would be difficult to interpret and could reflect chance, heterogeneity, or small-study effects rather than true publication bias. Consequently, the possibility of publication bias or selective non-publication cannot be excluded. Planned subgroup analyses by sex, age group, NMT content, dose, and adherence could not be performed because of the small number of included trials and incomplete reporting across studies. Consequently, potential effect modifiers could only be discussed narratively. Future trials should report intervention components, progression, adherence, and implementation fidelity in sufficient detail to permit more informative subgroup and dose-response analyses.

Despite these limitations, the use of exposure-adjusted incidence rate ratios, inclusion of large population-based trials, and a structured narrative synthesis for outcomes lacking adequate exposure data contribute to a fair assessment of the available evidence. Standardized neuromuscular training (NMT) protocols with well-defined content, intensity, and progression should be the main focus of future research to reduce heterogeneity and enhance trial comparability. Pooled analyses must be strengthened by exposure-adjusted reporting and consistent injury criteria. In particular, to prevent knee, ligament, and muscle injuries, longitudinal randomized controlled studies with adequate follow-up are required to determine the ideal NMT dosage and adherence criteria. To further understand differential impacts, research with sufficient power should provide subgroup analysis by age, sex, and competitive level. Monitoring implementation fidelity and adherence in practical contexts should also receive more attention. Lastly, conducting individual participant data meta-analyses and extending research to other geographic regions may help identify sources of variability and improve the generalizability of results.

## 5 Conclusion

This systematic review and meta-analysis suggest that neuromuscular training may reduce selected lower-limb injury rates among youth soccer athletes, with the most consistent protective signal observed for ankle injuries. However, the two outcomes that could be meta-analyzed, all lower-limb injuries and knee injuries, showed protective but non-significant pooled estimates. In addition, ankle, ligament, and muscle injury findings were limited by the availability of exposure-adjusted data from single trials and therefore require confirmation. Overall, the current evidence supports the potential value of NMT in youth soccer but does not establish a definitive preventive effect across all lower-limb injury categories. Larger randomized trials with standardized injury definitions, athlete-exposure reporting, and adherence monitoring are needed to clarify the magnitude and consistency of benefit.

The study is limited by high statistical heterogeneity, particularly in ankle and muscle injury data, reflecting the diverse methodologies across the included RCTs. Publication bias could not be formally assessed owing to the small number of contributing trials; therefore, selective non-publication cannot be excluded.

Future trials should adopt standardized soccer injury-surveillance and reporting methods, such as the consensus statement by Fuller et al. (13), to improve comparability across studies. Injury definitions, injury severity, exposure denominators, and athlete-exposure hours should be reported consistently across injury categories. In addition, NMT protocols should be described in sufficient detail, including exercise components, progression, session duration, frequency, adherence, and implementation fidelity. Such reporting would allow future reviews to perform more reliable exposure-adjusted pooling and subgroup analyses by age, sex, intervention dose, and NMT content.

## Data Availability

The original contributions presented in the study are included in the article/Supplementary Material, further inquiries can be directed to the corresponding author.
