# Supplementary material for: Preventive efficiency of neuromuscular training in reducing lower limb injuries among youth soccer athletes: a systematic review and meta-analysis of randomized controlled trials
Source: Front Sports Act Living. 2026 Jul 8;8:1853354. doi: 10.3389/fspor.2026.1853354 (PMC13387728; doi:10.3389/fspor.2026.1853354)
Supplement: Supplementary file 1 [file Table1.docx]

**Supplementary Table 1. Database-specific search strategy**

| **Database** | **Search string** | **Filters / limits** | **Date searched** |
| --- | --- | --- | --- |
| PubMed | (“soccer” OR “football” OR “soccer players”) AND (“neuromuscular training” OR NMT OR “balance training” OR “proprioceptive training” OR “stability exercises” OR “plyometric training”) AND (“injury prevention” OR “lower limb injury” OR “ACL injury” OR “ankle sprain” OR “hamstring strain”) | English; January 2000–October 2025; humans | October 2025 |
| Google Scholar | soccer OR football “neuromuscular training” “injury prevention” “youth” “lower limb injury” | English; January 2000–October 2025 | October 2025 |
| Cochrane Library | soccer OR football AND neuromuscular training AND injury prevention | January 2000–October 2025 | October 2025 |
| Embase | (‘soccer’ OR ‘football’) AND (‘neuromuscular training’ OR ‘balance training’ OR ‘proprioceptive training’ OR ‘plyometric training’) AND (‘injury prevention’ OR ‘lower limb injury’ OR ‘ACL injury’ OR ‘ankle sprain’ OR ‘hamstring strain’) | English; January 2000–October 2025; human | October 2025 |
